# Dataset on the optimization by response surface methodology for dried banana products using greenhouse solar drying in Thailand

**DOI:** 10.1016/j.dib.2023.109370

**Published:** 2023-07-05

**Authors:** Torpong Kreetachat, Saksit Imman, Kowit Suwannahong, Surachai Wongcharee, Trairat Muangthong-on, Nopparat Suriyachai

**Affiliations:** a School of Energy and Environment, University of Phayao Tambon Maeka, Amphur Muang, Phayao 56000, Thailand; b Faculty of Public Health, Burapha University, Tambon Saensuk, Amphur Muang, Chonburi 20131, Thailand; c Faculty of Engineering, Mahasarakham University, Tambon Khamriang, Amphur Kantarawichai, Mahasarakham 44150, Thailand; d Department of Environmental Engineering, Faculty of Engineering, King Mongkut's University of Technology Thonburi, Bangkok 10140, Thailand

**Keywords:** Greenhouse solar drying, Dried banana, Design optimization, Response surface methodology, Commercial use

## Abstract

The banana industry in Thailand holds immense potential, driven by favorable growing conditions, robust domestic consumption, and active participation in the export market. Solar dryers have the potential to revolutionize fruit processing by providing a sustainable, cost-effective, and nutritionally rich solution. This research aims to optimize the greenhouse solar drying process for bananas using response surface methodology. The specific variables under investigation are drying temperature and drying time. A designed greenhouse solar dryer, tailored for commercial use in the target area, was employed for the experiment. Statistical analysis and response surface methodology were utilized to evaluate the effects of the experimental variables on two key outputs: moisture content and color change of the dried banana product. The findings of this study contribute to a deeper understanding of the potential of solar drying in the context of banana processing. The research outcomes provide valuable insights for optimizing the solar drying process, thereby facilitating the development of the banana industry and its applicability.

**Specifications Table**

**Table:** 

Subject	Renewable Energy

Specific subject area	Greenhouse solar drying
Type of data	Raw data, Tables, Figures, and Graphs
How data were acquired	Experimental design for greenhouse solar drying of banana from commercial use. Analytical modeling and optimization using Statistica software
Data format	Raw and analyzed
Description of data collection	The samples were dried by greenhouse solar drying and characterized the quality of the product in terms of moisture content and color preservation. The moisture content was measured based on the relative weight. The color preservation was measured from the color measurement.
Data source location	Community enterprise area at Bantum, Phayao, Thailand (19°13′53.6''N 99°47′21.4''E).
Data accessibility	Repository name: Mendeley Data Data identification number: doi: 10.17632/87tfjn5s2h.1 Direct URL to data: https://data.mendeley.com/datasets/87tfjn5s2h

## Value of the Data

• The experiment was conducted using a designed greenhouse solar dryer with specific dimensions and components based on commercial use in the target area.
• The research emphasizes the significant variable of drying time and drying temperature on the quality of dried banana.
• Response surface methodology and statistical analysis were employed to evaluate the effects of experimental variables on moisture content and color change.
• The research contributes to understanding the potential of solar drying and its application in the context of banana processing.
• The research findings provide valuable insights for optimizing the solar drying process, contributing to the development of the banana industry and its agricultural sector.

## 1 Objective

The potential of bananas in Thailand is significant due to favorable growing conditions, strong domestic consumption, and active participation in the export market [1]. With continued support and investment, the banana industry is expected to thrive and contribute to the country's agricultural sector and economy. Currently, solar drying offers numerous benefits for dried bananas [2], [3], [4], including cost-effectiveness, nutritional retention, extended shelf life, convenience, sustainability, and income generation [5]. It is a viable and sustainable method for banana processing that adds value to the crop and contributes to the development of the agricultural sector. The objective of the research is to optimize the greenhouse solar drying process for bananas using response surface methodology. The specific variables to be optimized are the drying temperature and drying time. The research aims to provide statistical data and evaluate the effect of these variables on two key outputs: moisture content and color change of the dried banana product.

## 2 Data Description

**Fig. 1:**
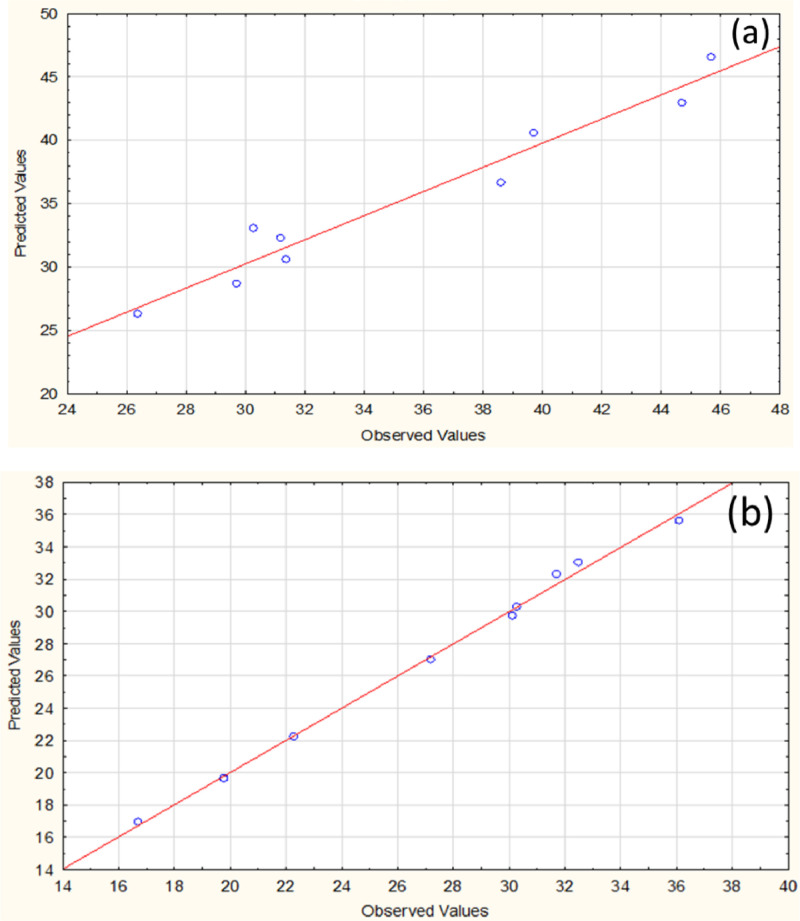
Actual and predicted response of respondents: Moisture content (a) and color change (b).

**Fig. 2:**
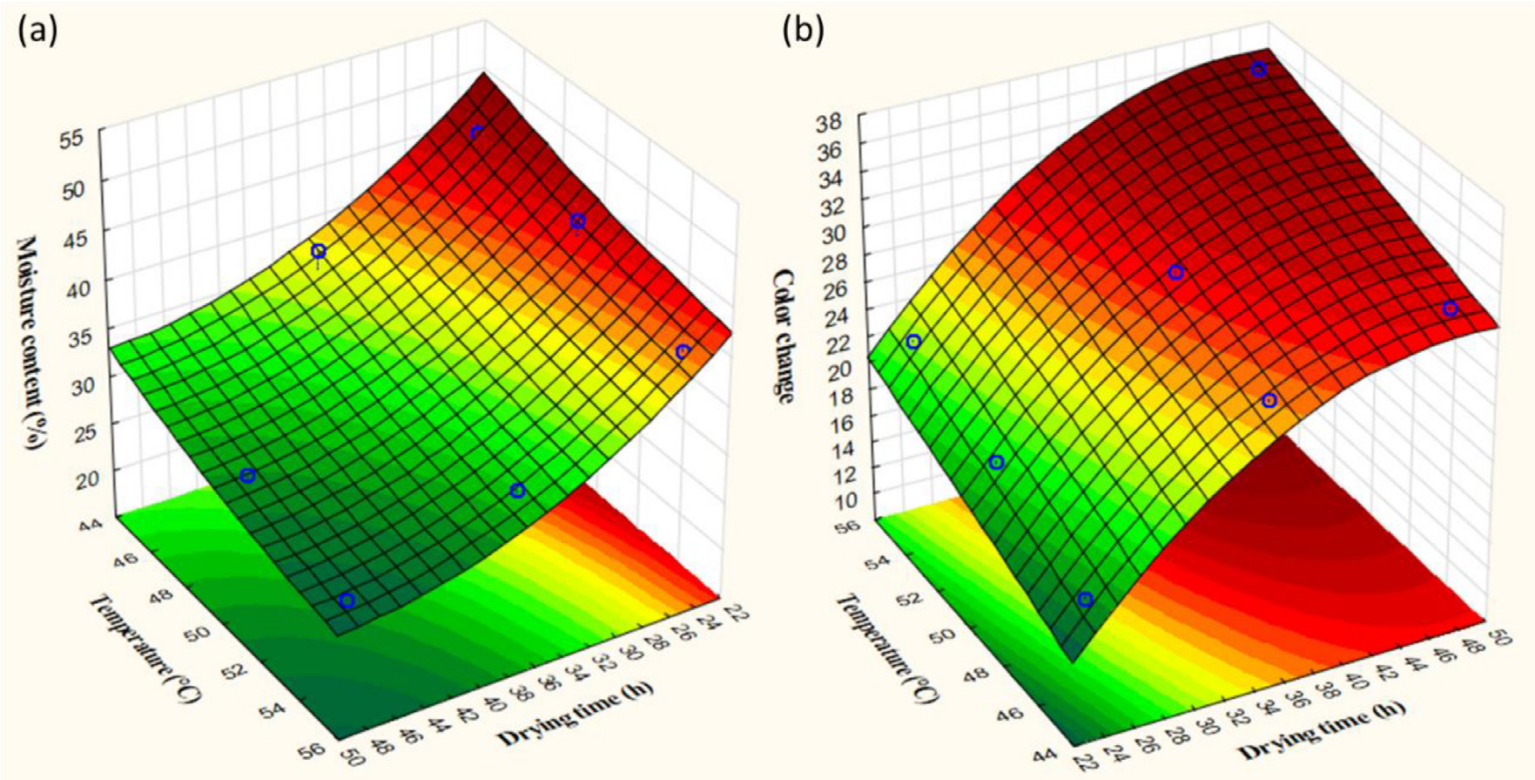
3D surface plot of moisture content (a) and color change (b).

**Fig. 3:**
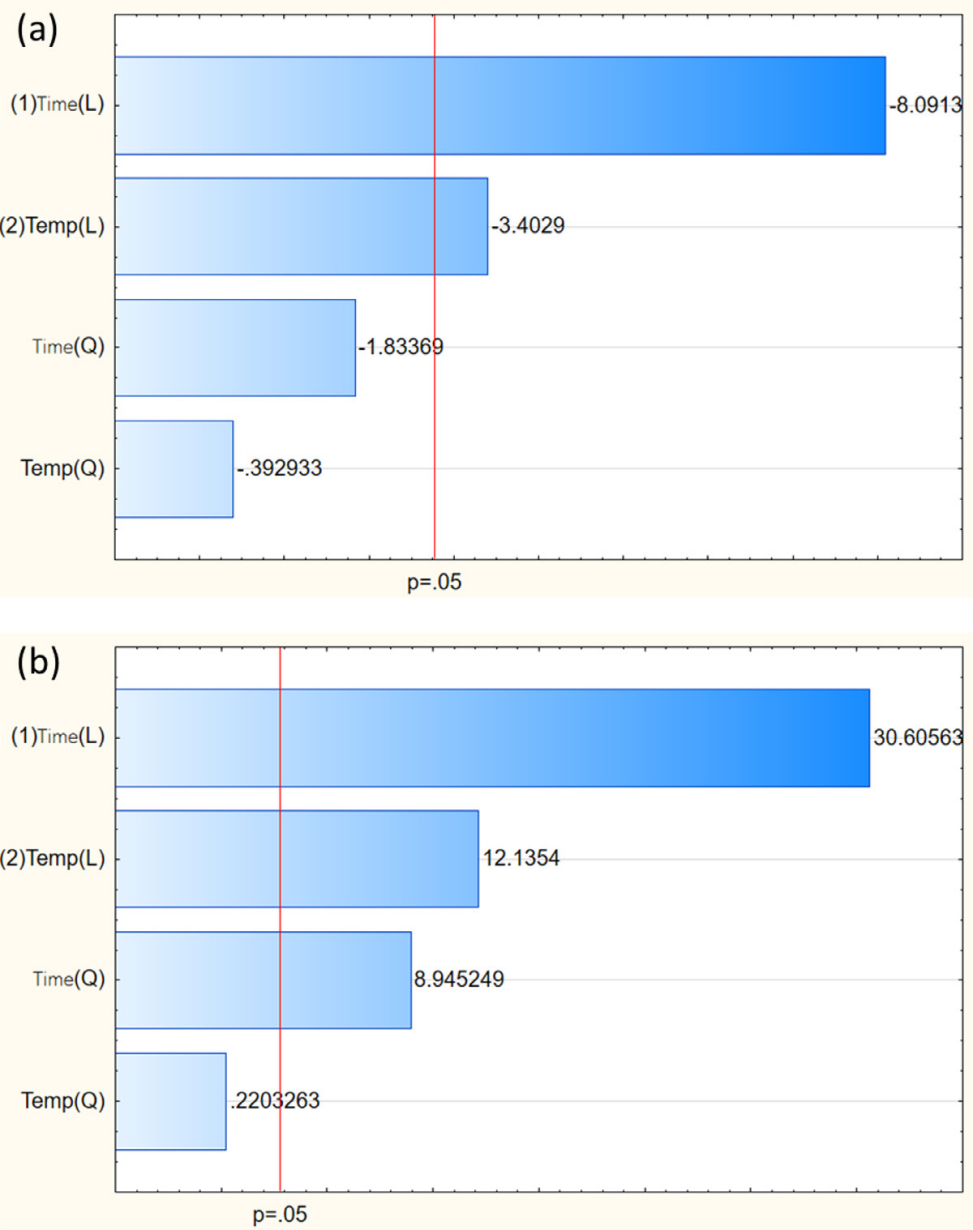
Pareto chart of standardized effects of moisture content (a) and color change (b).

Table 1 represents the coded value ranges of RSM based on full factorial design. The variables consist of drying time and drying temperature with three levels. The effect of experimental variables was evaluated based on the response of moisture content and color change. The summary of experimental results with 9 runs and 2 responses is shown in Table 2. The results of moisture and color change ranged from 45.7 – 26.4% and 16.7 – 36.1, respectively. The accuracy of responses was analyzed to clarify the fit of variance (Fig. 1). Model equations for all target responses with r-square are shown in Table 3. The models were statistically valid with the adjusted r-square of moisture content and color change of 90.54% and 99.31%, respectively. These showed the goodness of fit of both responses. The trend of relative results was illustrated based on the pattern of response surface methodology as shown in Fig. 2. In details, increasing temperature and drying time led to marked decreases in the moisture content, while showed an inversed trend in color change. Analysis of variance (ANOVA) was used to evaluate statistical significance of the model, as illustrated in Table 4 and 5. The p-value was used to evaluate the significance of each coefficient (p-value < 0.05). As the results, drying time showed a significant variable for moisture content, while both time and temperature affected the value of color change. According to Pareto chart of each response (Fig. 3), it was observed that drying time showed a higher influence on both responses than temperature. This could be described that the temperature range under the experiment was narrow due to the environmental condition during the experiment period. On the other hand, a wide range of drying times was designed based on the period of the working day. This study's final moisture content was similar to previous research on directly forced convection household solar dryers [5,6]. The case of sliced bananas in an active indirect mode solar dryer provided a final moisture content of 12% (wet basis) [3,7]. The difference in product quality could be influenced by the drying process design and the sample characteristics.

**Table 1:** Process parameters and their level based on full factorial design for solar drying.

Factors	-1	0	1
Drying time (hour)	24	36	48
Temperature (°C)	45	50	55

**Table 2:** Design matrix and responses.

Runs	Coded value		Actual value		Responses	
	Drying time (h)	Temp. (°C)	Drying time (h)	Temp. (°C)	Moisture content (%)	Color change (ΔE)
1	0	1	36	55	31.4	31.7
2	1	0	48	50	29.7	32.5
3	-1	-1	24	45	45.7	16.7
4	-1	0	24	50	44.7	19.8
5	1	1	48	55	26.4	36.1
6	0	-1	36	45	38.6	27.2
7	1	-1	48	45	31.2	30.3
8	-1	1	24	55	39.7	22.3
9	0	0	36	50	30.3	30.1

**Table 3:** Effect estimates for final regression model.

Responses	R^2^_value_	R^2^_(predicted)_	R^2^_(adjusted)_
Moisture content = 35.30000 - 14.26667A - 2.80000A^2^ - 6.00000B - 0.60000B^2^	99.84%	95.27%	90.54%
Color change = 27.41111 + 13.3666A +3.38333A^2^ + 5.30000B + 0.08333B^2^	99.98%	99.65%	99.31%

**Table 4:** ANOVA results for moisture content.

Factor	SS	df	MS	F	p
(1) Drying time (L+Q)	320.9867	2	160.4933	34.41601	0.003016
(2) Temperature (L+Q)	54.7200	2	27.3600	5.86705	0.064630
Error	18.6533	4	4.6633		
Total SS	394.3600	8

**Table 5:** ANOVA results for color change.

Factor	SS	df	MS	F	p
(1) Drying time (L+Q)	290.8956	2	145.4478	508.3612	0.000015
(2) Temperature (L+Q)	42.1489	2	21.0744	73.6583	0.000699
Error	1.1444	4	0.2861		
Total SS	334.1889	8

Furthermore, the greenhouse solar drying can have an effect on the nutritional values of the product, including protein, carbohydrates, naturally occurring sugars, fibers, and potassium. When conducted under controlled conditions, greenhouse solar drying helps retain the protein and carbohydrate contents of the product. Although there may be a minor degradation in naturally occurring sugars, the final content of total sugars increases due to the concentration of fruit during the drying process. The fiber and potassium content remains relatively unaffected by greenhouse solar drying [8,9]. Perspective in term of energy, the comparative energy consumption of greenhouse solar dryer and conventional drying (open sun, hot air, freeze, and microwave) can vary depending on several factors, including the specific drying technologies employed, the scale of drying operations, and the local climate conditions [10,11].

The data collected during the banana drying process include relevant parameters such as drying time and drying temperature. These parameters provide valuable information about the drying conditions and can be used to optimize and improve the drying process. It emphasizes the potential of the data to contribute to scientific knowledge, advance research, and support further investigations specifically related to banana drying. It had been suggested that maintaining a suitable range of moisture content and preserving the appearance of dried bananas requires a drying temperature above 50°C, along with a drying time longer than 36 hours. In term of rural applicability, the development of greenhouse solar drying in rural areas for commercial use has the potential to uplift local economies, empower farmers, and promote sustainable agricultural practices [12,13].

## 3 Experimental Design, Materials and Methods

### 3.1 Greenhouse Solar Dryer

**Fig. 4:**
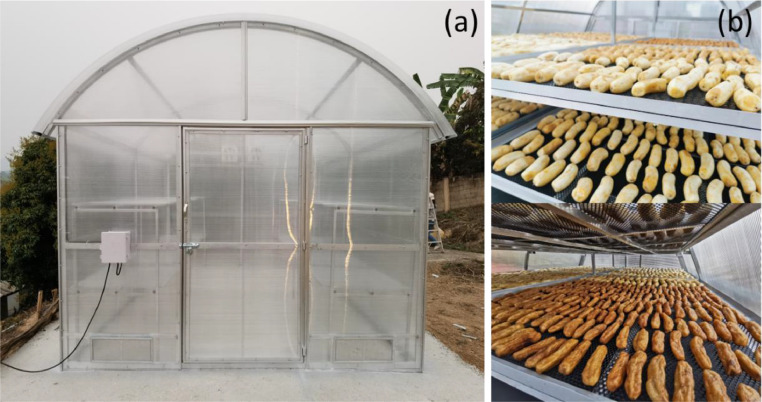
Photographs of greenhouse solar drying system (a) and samples of dried banana (b).

The solar dryer, located in the community enterprise area at Bantum, Phayao, Thailand (19°13′53.6''N 99°47′21.4''E), was designed, constructed, and tested (Fig. 4). It consists of various components, including a concrete floor, parabola dome, insulator, centrifugal ventilator, dryer shelf, and thermoregulatory system. The dryer's dimensions are 3 meters wide, 4 meters long, and 3 meters high. To provide transparency, a 6 mm thick polycarbonate sheet was used as the cover material, while the frame and housing were constructed using steel. The centrifugal ventilator, an axial fan operating at 220 volts and 1.2 amperes, allows for a theoretical air velocity of 1.5 m/s. Wind velocity was measured using a digital anemometer, and the temperature was monitored using a sensor with a range of 0-100°C and a precision of 0.1°C. Two shelves were evenly spaced in the drying zone to accommodate the drying process.

### 3.2 Sample Preparation

Fresh banana samples were collected from a local area within the Phayao province of Thailand. Prior to drying, the samples were thoroughly cleaned and peeled. The weight of the prepared samples was determined using an electronic weighing scale. Care was taken to place the samples in the dryer tray without overlapping to ensure uniform drying. At appropriate intervals, the products were removed from the tray and weighed. The drying experiment was carried out daily from 6:00 am to 6:00 pm, allowing 12 hours of working time per day until all experimental runs were completed. Throughout the experiment, the weather remained predominantly sunny, with no rain.

### 3.3 Determination of Moisture Content

The moisture content on the wet basis of the product was calculated according to Eq. (1):

(1) $$Moisture\;content\;\left(M_{w}\right)=\frac{W_{w}}{W_{w}+W_{d}}\;x\;100\%$$

where M_w_ = Moisture content on a percent basis (%); W_w_ = Total weight on wet weight (g); W_d_ = Total weight on dry weight (g)

### 3.4 Color Measurements

Surface color measurement was conducted using the L* a* b* system (Universal HunterLab, Model 45/0 S/N CX- 0413), calibrated to a standard white tile (L*=91.7, a*=-1.16, b*=1.06). L*corresponds to lightness, a* represents red (+)/green (-), and b* refers to yellow (+)/blue (-). A total of three measurements were carried out for each treatment. Color measurements such as chroma and hue angle have been proposed as more practical [14]. The overall color change or difference (ΔE) from the fresh to dried sample was also calculated using the equation below:

(2) $$\Delta E=\sqrt{{\left(L_{0*}-L^{*}\right)}^{2}+}{\left(a_{0*}-a^{*}\right)}^{2}+{\left(b_{0*}-b^{*}\right)}^{2}$$

where, *L*0*, a0* and *b*0*, are the L*, a*, and b* values of the fresh sample and L*, a*, and b* are corresponding values of the dried sample, respectively. A larger ΔE denotes greater color change from the fresh sample.

## 4 Experimental Design

In this study, the effect of the selected condition was designed and studied using a full factorial design. The RSM was used to investigate the impact of drying variables. The experimental condition comprised 9 combinations with drying time (24 – 48 hours) and drying temperature (45 – 55°C). The model was evaluated based on the response of moisture content and color change.

## Ethics Statements

This work did not involve the use of animal or human subjects. The data did not encounter any ethical issues and data gathered using social media.

## CRediT authorship contribution statement

**Torpong Kreetachat:** Conceptualization, Methodology, Formal analysis, Writing – original draft. **Saksit Imman:** Investigation, Writing – review & editing. **Kowit Suwannahong:** Investigation, Validation. **Surachai Wongcharee:** Methodology, Validation. **Trairat Muangthong-on:** Methodology, Validation. **Nopparat Suriyachai:** Conceptualization, Methodology, Formal analysis, Writing – review & editing, Supervision.

## Declaration of Competing Interest

The authors declare that they have no known competing financial interests or personal relationships that could have appeared to influence the work reported in this article.

## Data Availability

Data from greenhouse solar drying using response surface methodology for the production of dried banana (Original data) (Mendeley Data).
